# Sample size used to validate a scale: a review of publications on newly-developed patient reported outcomes measures

**DOI:** 10.1186/s12955-014-0176-2

**Published:** 2014-12-09

**Authors:** Emmanuelle Anthoine, Leïla Moret, Antoine Regnault, Véronique Sébille, Jean-Benoit Hardouin

**Affiliations:** Public Health Department, University Hospital of Nantes, 85, rue Saint Jacques, 44093 Nantes Cedex 1, France; EA 4275 SPHERE “bioStatistics, Pharmacoepidemiology and Human sciEnces Research tEam”, University of Nantes, 1, rue Gaston Veil, 44035 Nantes Cedex 1, France; Biometry Platform, University Hospital of Nantes, 5, Allée de l’Ile Gloriette, 44093 Nantes Cedex 1, France; Mapi HEOR & Strategic Market Access, 27 rue de la Villette, 69003 Lyon, France

**Keywords:** Psychometrics, Review, Sample size, Scale, Health status, Validation

## Abstract

**Purpose:**

New patient reported outcome (PRO) measures are regularly developed to assess various aspects of the patients’ perspective on their disease and treatment. For these instruments to be useful in clinical research, they must undergo a proper psychometric validation, including demonstration of cross-sectional and longitudinal measurement properties. This quantitative evaluation requires a study to be conducted on an appropriate sample size. The aim of this research was to list and describe practices in PRO and proxy PRO primary psychometric validation studies, focusing primarily on the practices used to determine sample size.

**Methods:**

A literature review of articles published in PubMed between January 2009 and September 2011 was conducted. Three selection criteria were applied including a search strategy, an article selection strategy, and data extraction. Agreements between authors were assessed, and practices of validation were described.

**Results:**

Data were extracted from 114 relevant articles. Within these, sample size determination was low (9.6%, 11/114), and were reported as either an arbitrary minimum sample size (n = 2), a subject to item ratio (n = 4), or the method was not explicitly stated (n = 5). Very few articles (4%, 5/114) compared *a posteriori* their sample size to a subject to item ratio. Content validity, construct validity, criterion validity and internal consistency were the most frequently measurement properties assessed in the validation studies.

Approximately 92% of the articles reported a subject to item ratio greater than or equal to 2, whereas 25% had a ratio greater than or equal to 20. About 90% of articles had a sample size greater than or equal to 100, whereas 7% had a sample size greater than or equal to 1000.

**Conclusions:**

The sample size determination for psychometric validation studies is rarely ever justified *a priori*. This emphasizes the lack of clear scientifically sound recommendations on this topic. Existing methods to determine the sample size needed to assess the various measurement properties of interest should be made more easily available.

**Electronic supplementary material:**

The online version of this article (doi:10.1186/s12955-014-0176-2) contains supplementary material, which is available to authorized users.

## Introduction

Measuring patient reported outcomes (PRO) has become a common clinical practice. This is primarily because a patient’s perspective on their health is central to a number of conditions, and because patients have become more forthcoming in describing their health status and illness experience. PRO measurements can facilitate patient involvement in decision-making about their own care, and may help healthcare professionals to identify patients concerns. This measurement is also essential in clinical research, as PROs are frequently used as study endpoints. As a consequence, new PRO measures are now regularly developed.

Prior to using PRO measures in clinical practice or research, instruments need to be developed and validated cautiously, in order to avoid biased results that might lead to incorrect interpretations. The development process of a PRO is fairly well defined [1,2]. The development stage for a PRO questionnaire, as proposed by Fayers and Machin [2], include generating an initial hypothetical model, defining the target population, generating items by qualitative methods, followed by pre-testing and field-testing the questionnaire. The validation stage aims to assess the measurement properties of the PRO measure. This includes the assessment of validity (content validity, face validity, construct validity and criterion validity), reliability (repeatability and internal consistency) and responsiveness. This psychometric validation step is very important for a new PRO measure to be accepted and widely used [1,2].

Sample size is recognized as a key parameter for the planning of studies in many areas of clinical research. This is exemplified by its use in a growing number of published guidelines including: CONSORT (CONsolidated Standards Of Reporting Trials) [3], STROBE (STrengthening the Reporting of OBservational studies in Epidemiology) [4], TREND (Transparent Reporting of Evaluations with Nonrandomized Designs) [5], STARD (STAndards for the Reporting of Diagnostic accuracy studies) [6], STREGA (Strengthening the reporting of genetic association studies) [7], as well as in the recently published CONSORT PRO [8].

Nevertheless, sample size is only briefly mentioned in most guidelines published in order to help researchers design studies aimed at assessing PRO psychometric properties, or evaluating the methodological quality of those studies [1,9-11]. Moreover, the International Society for Quality of Life research (ISOQOL) recently defined minimum standards required for the design and selection of a PRO measure but did not mention sample size determination [12]. Although inappropriate sample size can lead to erroneous findings in many aspects of PRO development and validation, in particular the identification of the correct structure of the questionnaire (eg. number of dimensions and items in each dimension), no consensus exists to define sample size with the same rigour as found in most clinical research based on clinical or biological criteria (eg. arbitrarily determined sample size or subject to item ratio).

Our motivation was to examine how developers of new PRO measures currently determine their sample size, and report the critical steps of psychometric validation of their newly developed instruments, in terms of design, measurement properties, and statistical methods. To our knowledge, the last review aimed at investigating the methodology used in the construction of a measurement scale was reported in 1995 [13].

The aim of the study was to perform a comprehensive literature review to enable the practices in PRO primary psychometric validation studies to be listed and described, with a particular focus on the importance on sample size determination.

## Materials and methods

A literature review was conducted from September 2011 to September 2012, on articles published between January 2009 and September 2011, following the Centre for Review and Dissemination’s (CRD) guidelines for undertaking reviews in health care [14], and recommendations published by Mokkink [15]. It comprised three stages:

- Search strategy: Identification of articles by specifying inclusion and exclusion criteria, keywords and search strings in the PubMed database.
- Selection: Article pre-selection by reading titles, followed by a selection by reading abstracts.
- Extraction: Extraction of data from articles, and filling in a reading grid and providing a synthesis.

### Psychometric properties definitions (Table 1)

**Table 1:** **Psychometric properties definitions in the field of health-related assessment**

***Properties***	***Definitions***
Content validity	The ability of an instrument to reflect the domain of interest and the conceptual definition of a construct. In order to claim content validity, there is no formal statistical testing, but item generation process should include a review of published data and literature, interviews from targeted patients and an expert panel to approach item relevance [2].
Face validity	The ability of an instrument to be understandable and relevant for the targeted population. It concerns the critical review of an instrument after it has been constructed and generally includes a pilot testing [2].
Construct validity	The ability of an instrument to measure the construct that it was designed to measure. A hypothetical model has to be formed, the constructs to be assessed have to be described and their relationships have to be postulated. If the results confirm prior expectations about the constructs, the instrument may be valid [2].
Convergent validity	Involves that items of a subscale correlate higher than a threshold with each other, or with the total sum-score of their own subscale [2].
Divergent validity	Involves that items within any one subscale should not correlate too highly with external items or with the total sum-score of another subscale [2].
Known group validity	The ability of an instrument to be sensitive to differences between groups of patients that may be anticipated to score differently in the predicted direction [2].
Criterion validity	The assessment of an instrument against the true value, or a standard accepted as the true value. It can be divided into concurrent validity and predictive validity [2]*.*
Concurrent validity	The association of an instrument with accepted standards [2].
Predictive validity	The ability of an instrument to predict future health status or test results. Future health status is considered as a better indicator than the true value or a standard [2].
Reliability	Determining that a measurement yields reproducible and consistent results [2].
Internal consistency	The ability of an instrument to have interrelated items [2].
Repeatability	(Test-retest reliability) The ability of the scores of an instrument to be reproducible if it is used on the same patient while the patient’s condition has not changed (measurements repeated over time) [2]. *Measurement error* is the systematic and random error of a patient’s score that is not attributed to true changes in the construct to be measured [17].
Responsiveness	The ability of an instrument to detect change when a patient’s health status improves or deteriorates [2].

The definition of the psychometric properties was defined by the individual investigators in a consensual manner prior to beginning the review. This is important because experts often employ different terminologies and definitions for the same concept [1,2,9,10,12,16,17]. Standard references of psychometric theory in the field of health-related assessment were used to define the psychometric properties that were collected [2,17].

### Literature review

The authors, including three statisticians (EA, JBH, VS) and a public health physician (LM), took part in the literature review, and were responsible for designing and performing the search strategy, article selection and data collection.

#### Stage 1: Search strategy

The primary inclusion and exclusion criteria were chosen to meet the objective of the study: to examine how many individuals are included in PRO validation studies, and how developers of PRO measures report the steps involved in psychometric validation, including sample size determination. Because the focus was on primary studies, we excluded studies that reported translation and transcultural validation, revised scale validation and scale revalidation.

Inclusion criteria were:

- Measure of a patient reported outcome (PRO)
- Report of a scale construction and evaluation of its psychometric properties (primary study)
- Published in English or French
- Published from January 2009 to September 2011
- Abstracts available on PubMed
- Report of psychometric properties validation
- Exclusion criteria were:
- Instruments with a predominantly diagnostic, screening or prognostic purpose
- Systematic review articles
- Comparisons of scale psychometric properties
- Transcultural adaptation and translation validation studies
- Studies using a scale without performing any validation
- Symptom inventory
- Scale revalidation on another sample or deepening of scale psychometric properties
- Short or revised form of a scale
- Articles exclusively related to content and face validation.

The PubMed database was searched for relevant articles as it is the main medical database and because we focus our attention on PRO. Because the use of searchable technical terms for indexing international literature in databases is not always up-to-date, we defined a search strategy composed of free text terms, synonyms and MeSH terms with high sensitivity but low specificity. The search included the terms or expressions “score”, “scale”, “index”, “indicator”, “outcome”, “composite”, “construction”, “development”, “item selection”, “validation”, and “questionnaire”, but excluded the terms or expressions “translation”, “transcultural” and “cross-cultural”. The search string is provided in the Appendix 1.

#### Stage 2: Article selection

To pre-select articles, EA reviewed the titles of all records retrieved from the initial search. LM, JBH and VS then performed an independent review of the same articles by evenly sharing the full list of titles. Inclusion or exclusion disagreements were resolved by a third reviewer (e.g.: disagreements between EA and LM, on the titles they both read, were resolved through JBH). Once articles were pre-selected by title, the same procedure was used to score the available abstracts, using the same article selection and disagreement resolution process. There were two kinds of disagreements: those related to inclusion or exclusion of articles and those related to the reason of exclusion.

#### Stage 3: Data extraction

The number of articles remaining after the second stage was still fairly large. In order to proceed with a manageable data extraction phase, in terms of time and available resources, whilst keeping the data representative of the literature, a sample of articles was randomly selected (Additional file 1 (AF1)), using the sample function in R 2.12.1. The data from these articles was extracted and uploaded to the reading grid by EA. In addition, LM, JBH and VS each reviewed 10 randomly selected articles independently from EA.

The extraction reading grid was formulated based on psychometric properties definitions from the standard references [2,17]. The variables of the grid were discussed among the authors and yielded 60 variables in 5 domains (general information on article, study and scale, determination of sample size, items distribution and evaluation of psychometric properties) to describe the reporting of studies in terms of design, measurement properties and statistical methods (Additional file 2 (AF2)).

### Statistical analysis

To evaluate whether the reviewers agreed with each other, the proportion of observed agreements P_0_, and the Kappa were computed. This allowed the judgement consistency related to inclusion and exclusion of articles to be measured, in both the pre-selection step, and the subsequent selection step [18].

Descriptive statistical analyses (mean, standard error and frequencies) for each variable of the extraction reading grid were performed.

The software R 2.12.1 was used.

## Results

### Article selection

**Figure 1:**
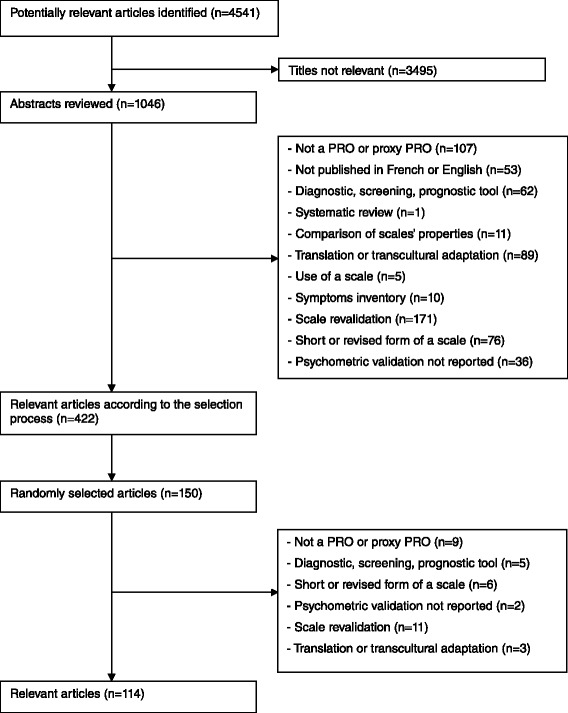
**Flow chart of selection process.**

The search string identified 4541 potentially relevant articles published from January 2009 to September 2011. After the pre-selection step, 1046 articles were selected (Figure 1). The proportion of observed agreements P_0_ between EA and three other authors ranged from 88 to 93%, and Kappa coefficients from 0.76 to 0.86.

After the selection step, 422 articles were included. The proportion of observed agreements P_0_ ranged from 79 to 82% and Kappa coefficients from 0.80 to 0.86. The article exclusion criteria most frequently encountered included: secondary validation of the scale, transcultural adaptation or translation, short form and non PRO or proxy PRO.

Due to the large number of articles, data were extracted from 150 randomly selected articles, with 36 being excluded according to exclusion criteria (Figure 1), resulting in 114 publications.

### Practices in PRO primary psychometric validation studies

The list of the 114 randomly selected articles, from which the results were obtained, is provided as AF1.

#### General description of articles, studies and scales (Table 2)

**Table 2:** **General description of journals and scales**

***n = 114***		
**Journals**		
Main topic of journal	Clinical	66.7% (76)
	Methodological	8.8% (10)
	Psychological	17.5% (20)
	Other	7.0% (8)
Impact factor	Mean (SD); median; range	2.53 (1.55); 2.25; [0.38; 11.01]
**Scales**		
Concept of interest	Quality of life	29.8% (34)
	Behavior/Attitude	21.1% (24)
	Social psychological functioning	15.8% (18)
	Satisfaction	13.1% (15)
	Symptom severity	10.6% (12)
	Knowledge/Literacy	6.1% (7)
	Physical functioning	3.5% (4)
Number of dimensions	Mean (SD); median; range	3.7 (2.7); 3; [1; 13]
Number of items per dimension	Mean (SD); median; range	11.8 (32); 7; [1; 340]
Type of measurement scale	Dichotomous	2.6% (3)
	Likert ordinal	84.2% (96)
	Nominal	3.5% (4)
	Numeric rating scale	6.2% (7)
	Several kinds	0.9% (1)
	Not mentioned	2.6% (3)

Data are percentages (n) and otherwise indicated.

Standard deviation (SD).

A wide range of concepts were investigated. Quality of life and behaviour were the main studied (50.9%, n = 58). Scales were mainly published in clinical journals (66.7%, n = 76), developed in English (62.3%, n = 71), and were more often specific (72.8%, n = 83). The median number of dimensions per scale was 3 [min = 1; max = 13], and the median number of items per dimension was 7 [min = 1; max = 340]. The 340 items scale was an occupational-based child developmental scale. The Likert ordinal scale was mostly used (84.2%, n = 96), and 78.1% of scales (n = 89) were multidimensional.

#### Practices of sample size determination (Table 3)

**Table 3:** **Sample size determination**

***n = 114***		
Sample size included	Mean (SD); Median; range	509 (1094); 207; [24; 7906]
Subject to item ratio	Mean (SD); Median; range	28 (67); 10; [1; 527]
*A priori* determination of the required sample size	Yes	9.6% (11)
	Justification *a posteriori*	4.4% (5)
	No	86.0% (98)

Standard deviation (SD).

The median sample size was 207 patients [min = 24; max = 7906] and the determination of sample size was justified in less than 10% (n = 11) of the articles. In 5 papers, the method used to define the sample size was not explicitly stated and only references were provided. In 6 papers the reported methods for sample size determination were, either an arbitrary minimum sample size according to a methodological reference used by the authors (n = 2), or the subject to item ratio (n = 4), which is the frequently recommended approach when performing an exploratory factor analysis (EFA). However, the subject to item ratio varied from 1.2 to 10 according to references used by the authors. Finally in 2 papers, a sample size was computed for detecting a difference in two groups of patients, or for having a high correlation between a reference and a new scale. Five articles (4%) compared their number of included patients to a subject to item ratio *a posteriori*, from 5 to 20, to justify their sample size.

Approximately 17% of the publications discussed the impact of sample size. In half of these, it was noted that the sample size was too small, for example, “One possible limitation is the relatively small number of patients. But a sample size of approximately 100 subjects is considered a minimum when conducting multivariate analyses” (reference 31 in AF1), “The sample size for exploring the preliminary validity of the questionnaire probably does not have sufficient statistical power” (reference 46 in AF1), “In the light of the sample size, the data should be interpreted with caution” (reference 68 in AF1), “We obtained a ratio of 3.94 respondents for each question, which might be a limitation for the factor analysis” (reference 9 in AF1). In these studies, the median sample size was 121, with a minimum of 30 and a maximum of 725, and the median subject to item ratio was 4 with a minimum of 1 and a maximum of 26.

For other articles the sample size was discussed as being adequate, e.g. “The relatively small number of participants limits the robustness of the computation of the factor structure, but the results are nevertheless acceptable, with the number of respondents being five times the number of items in the analysis” (reference 17 in AF1), “Exploratory factor analysis results showed that all items had high communalities, which substantially reduces the influence of sample size on exploratory factor analysis results” (reference 55 in AF1). In these studies, the median sample size was 191, with a minimum of 63 and a maximum of 422, and the median subject to item ratio was 6 with a minimum of 1 and a maximum of 28.

Few articles mentioned that there was no consensus as to how to compute a required sample size for validating a PRO: “Approaches to determining sample size adequacy vary considerably by type and purpose of analysis” (reference 21 in AF1).

**Figure 2:**
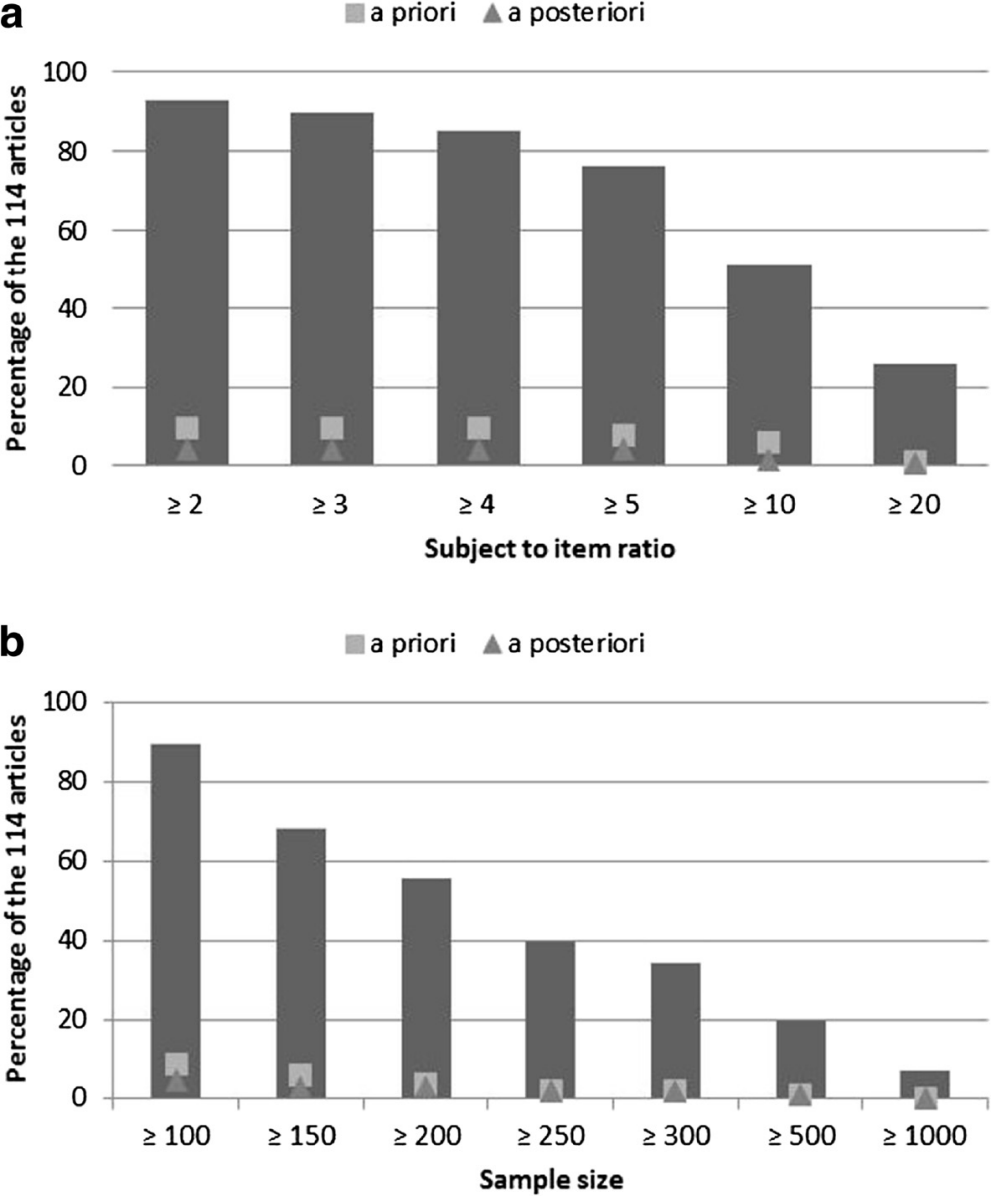
**Repartition of the articles according to thresholds recommended in the literature. a**: According to thresholds of subject to item ratio. **b**: According to thresholds of sample size.

Sample sizes of our 114 reviewed articles were then compared using recommendations of the literature in terms of subject to item ratio and absolute minimum sample size (Figure 2). *A priori* means that articles, where the sample size was determined *a priori,* were considered and *a posteriori* means that articles, where the sample size was determined *a posteriori,* were considered. In the reviewed articles of the present study, the mean subject to item ratio was 28 and the median was 11, with a minimum of 1 and a maximum of 527. About 92% of the articles displayed a subject to item ratio ≥2, whereas 25% had a ratio ≥20. About 90% of the articles had a sample size ≥100, whereas 7% had a sample size ≥1000. Among the 114 articles, 1% have determined an *a priori* sample size with the rule of the ratio equal to 20, and 2% with the guidance 300 = good.

#### Practices of the psychometric properties’ evaluation

Although our focus was on sample size determination, the practices of validation were also studied.

Missing values rates were reported in 22% (n = 25) of studies. Of these, 32% (n = 8) reported high missing value rates for at least one item, and reported that they eliminated items with high missing value rates (mean rate of 18% reported). Ceiling and floor effects were evaluated in 35% (n = 40) of articles, whereas items or score distributions were more often (61.4%, n = 70) assessed.

mong the 89 multidimensional scales (78.1%), a global score was computed in 65.2% (n = 58). In 81% (n = 47) of these, the justification to calculate a global score was not given. In almost half of the papers, the scoring method was not mentioned.

Face validity (65.8%, n = 75) was explored less often than content validity (94.7%, n = 108). Criteria validity was often evaluated (70.2%, n = 80), but most of articles (98.7%, n = 79) assessed concurrent validity, whereas 3.7% (n = 3) assessed predictive validity.

At least one aspect of construct validity was evaluated in 90.3% (n = 103) of the articles. Convergent validity (84.5%, n = 87), EFA (79.6%, n = 82) and known group validity (57.3%, n = 59) were the most explored, whereas divergent validity (17.5%, n = 18) and confirmatory factor analysis (CFA) (15.5%, n = 16) were the least. Before confirming the structure of the questionnaire by a CFA, it was predefined by EFA in 87.5% (n = 14) of the studies.

More than half of the studies (n = 69) explored repeatability. The intraclass correlation coefficient (ICC) and Pearson correlation coefficient were the two most common methods used, and were reported in 52.2% (n = 36) and 49.3% (n = 34) of the articles respectively. For a large majority of the scales (89.5%, n = 102), internal consistency was assessed. Most of them (95.1%, n = 97) used a Cronbach α coefficient. Responsiveness was rarely appraised (10.5%, n = 12), and was mostly assessed by a paired t test (75%, n = 9).

Tables are presented in AF2.

## Discussion

This literature review aimed to describe validation practices, with a primary focus on sample size, and focussed on 114 psychometric validation studies of new PRO measures, published between January 2009 and September 2011. The process of validation requires collecting a comprehensive body of evidence on the measurement properties of a scale including content and face validity, construct validity, criterion validity, and reliability and responsiveness. Numerous literature reviews, aimed at describing psychometric properties of scales, exist but they are limited to a specific disease, with the objective of comparing and choosing the appropriate instrument [19-23]. To our knowledge only one review, dating from 1995, aimed to investigate the methodology used in the construction of a measurement scale and proposed recommendations [13]. However, given the widespread use of PRO measures, it is therefore of interest to obtain a clear picture of how these measures are currently validated, and especially how sample size is planned.

Results of the review revealed that the method used for the sample size determination was defined *a priori* in less than 10% of articles. Four per cent of articles compared the numbers of included patients to a subject to item ratio *a posteriori*, to justify their sample size. Thus, 86% of the validation studies didn’t provide any robust justification for the sample size included. This high rate is of concern, because determining a sample size is required to achieve a given precision, or to have enough power to reject a false null hypothesis while being confident in this result. It is therefore of interest to motivate researchers to control the type II error, or to think *a priori* about the precision they want to have, before testing the null hypothesis regarding the structure of a scale. The lack of consensus regarding how to compute the sample size was pointed out in two papers of the review [24,25]. Indeed, subject to item ratio is a frequently used method to determine a required sample size to perform an EFA, but with various recommendations. For several authors, this ratio is partly determined by the nature of the data, i.e. the stronger the data, the smaller the sample size can be. Strong data display uniformly high communalities without cross-loadings [26]. Recommendations range from 2 to 20 subjects per item [27,28], with an absolute minimum of 100 to 250 subjects [29-31]. Comrey and Lee [32] provided the following guidance: 100 = poor, 200 = fair, 300 = good, 500 = very good, ≥1000 = excellent. In the articles reviewed in this study, the mean subject to item ratio was 28, with a minimum of 1 and a maximum of 527.

Recommendations in the literature for the sample size determination when conducting a CFA are also disparate (ranging from 150 to 1000 subjects), and seem to depend on the normality of data, and parameter estimation methods [33-36]. Some authors suggested two different sample sizes planning methods when performing a CFA. MacCallum et al. [37] suggested, in 1996, a method to determine a minimum sample size required to achieve a given level of power, for a test of fit using the RMSEA fit index. More recently, Lai and Kelley [38] developed a method to obtain sufficiently narrow confidence intervals for the model parameters of interest. These methods seem to be unused by PRO developers.

Moreover, whether it is used for performing an EFA or a CFA, most of published recommendations don’t express their opinion on the issue of sample size [1,9-12], which doesn’t facilitate good practice. For example, the COSMIN (COnsensus-based Standards for the selection of health Measurement INstruments) group assessed if the included sample size was “adequate” [11], but did not define its meaning or interpretation, and the Scientific Advisory Committee of the Medical Outcomes Trust noted that developers should include methods of sample size determination [9].

The current absence of clear guidance and the lack of consensus about how to compute *a priori* sample size are two key issues of sample size determination.

Several technical pitfalls in the psychometric validation were also highlighted. The first one pertains to the fact that descriptive information about items and score distributions were rarely given, while they are important in our opinion. For example, missing value rate was evaluated in only 22% of the studies, but an item with a lot of missing values is probably not relevant or understandable for patients.

The second one deals with content validity. It is encouraged to involve patients during the development phase of the instrument, in order to ensure content validity of a PRO measure, and to represent patient values [39]. This is particularly central in the Food and Drug Administration guidance [1] and this recommendation has to be supported. However, our literature review showed that patients were less often asked for interviews or focus groups than experts, whereas they are in the best position to describe their illness experience.

Finally, CFA was seldom (16%) performed for the study of construct validity. In the framework of a CFA, hypothesis of relationships between items and factors, and between factors, have to be postulated [33] and, once a hypothesized model is established, a CFA will confirm that it provides a good fit to the observed data. This makes CFA a method that is probably better suited than EFA for validation of instruments with a predefined measurement model. The practice of defining the structure during the development phase of a PRO measure should be followed, but was mentioned in only 2 of the reviewed papers.

Our research has some limitations. The first one relates to the absence of unique reference terminologies and definitions of measurement properties. This made the standardized extraction of data challenging. Mokkink [17] confirmed this by concluding that the correct answer probably doesn’t exist. We selected two references in the field of health-related assessment [2,17] and tried to be as clear as possible, so that readers understood the concepts that were explored. The second limitation relates to the fairly short publication period included in our literature search. This was a deliberate decision. We anticipated that even in a short period, many publications would be included, and this was confirmed by the retention of 422 relevant articles using our selection process. This prompted us to use a reductive random selection step to make the data extraction phase manageable, whilst keeping the results representative of the targeted literature, and representative of current practices in terms of psychometric validation. Indeed, there is no reason that an important change in practices would have happened as no recommendation in terms of sample size determination was published since 2011. It should be noted that we deliberately included only publications on the primary validation of PRO measures. Indeed, validation of PRO measures (for new linguistic versions of an existing PRO measure or a validation in another population) involves slightly different questions and would not necessarily compare with primary validation. Hence, we preferred to not include those. Another possible limitation was that only the PubMed database was used, but we were specifically interested in psychometric validation practices in the medical field. Finally, only articles published in English or French were included, as none of the authors were fluent in other foreign languages.

## Conclusion

Since sample size determination is hardly ever justified *a priori* in these studies, work still has to be done to make sure that validation studies of PRO measures are performed on a proper number of patients.

Clear and scientifically sound recommendations on the sample size for validation studies remain to be developed. These recommendations should probably depend on the methods envisaged for the assessment of measurement properties but they certainly must be based on rigorous evidence, which may be generated by formal calculations or simulation studies. Such recommendations would be helpful to PRO researchers designing validation studies and would warrant that new PRO measures are appropriately validated, with enough patients involved in the assessment of their measurement properties.

## Appendix 1: Search string

(score$ OR scale$ OR index$ OR indicator$ OR outcome$ OR composite$)

AND

(construction$ OR development$ OR “item selection”, OR valid$)

AND

(questionnaire$)

NOT

(translation$ OR transcultural$ OR “cross-cultural”)

## Additional files

Additional file 1:
**Literature review articles.** The list of the 114 reviewed articles. Additional file 2:
**Practices of the psychometric properties’ evaluation.** Tables.
