# Supplementary material for: Sample size used to validate a scale: a review of publications on newly-developed patient reported outcomes measures
Source: Health Qual Life Outcomes. 2014 Dec 9;12:2. doi: 10.1186/s12955-014-0176-2 (PMC4275948; doi:10.1186/s12955-014-0176-2)
Supplement: Additional file 1: — Literature review articles. The list of the 114 reviewed articles. [file 12955_2014_176_MOESM1_ESM.pdf]

How many individuals are used to validate a scale? A review of publications on newly-developed patient reported outcomes measures

Emmanuelle Anthoine, Leïla Moret, Antoine Regnault, Véronique Sébille, Jean-Benoît Hardouin

Systematic literature review articles

1. AbuRuz SM, Bulatova NR, Tawalbeh MI: **Development and validation of the Arabic allergic rhinitis quality of life questionnaire.** *Saudi Med J* 2009, **30**:1577–83.

2. Agarwal R: **Developing a self-administered CKD symptom assessment instrument.** *Nephrol Dial Transplant* 2010, **25**:160–6.

3. Aletras VH, Kostarelis A, Tsitouridou M, Niakas D, Nicolaou A: **Development and preliminary validation of a questionnaire to measure satisfaction with home care in Greece: an exploratory factor analysis of polychoric correlations.** *BMC Health Serv Res* 2010, **10**:189.

4. Al-shair K, Kolsum U, Berry P, Smith J, Caress A, Singh D, Vestbo J: **Development, dimensions, reliability and validity of the novel Manchester COPD fatigue scale.** *Thorax* 2009, **64**:950–5.

5. Althof SE, Perelman MA, Rosen RC: **The Subjective Sexual Arousal Scale for Men (SSASM): preliminary development and psychometric validation of a multidimensional measure of subjective male sexual arousal.** *J Sex Med* 2011, **8**:2255–68.

6. Andersson O, Rydén A, Ruth M, Möller RY, Finizia C: **Development and validation of a laryngopharyngeal reflux questionnaire, the Pharyngeal Reflux Symptom Questionnaire.** *Scand J Gastroenterol* 2010, **45**:147–59.

7. Bieber C, Müller KG, Nicolai J, Hartmann M, Eich W: **How does your doctor talk with you? Preliminary validation of a brief patient self-report questionnaire on the quality of physician-patient interaction.** *J Clin Psychol Med Settings* 2010, **17**:125–36.

8. Boiko O V, Baker SR, Gibson BJ, Locker D, Sufi F, Barlow APS, Robinson PG: **Construction and validation of the quality of life measure for dentine hypersensitivity (DHEQ).** *J Clin Periodontol* 2010, **37**:973–80.

9. Borges MC, Ferraz E, Pontes SMR, Cetlin A de CVA, Caldeira RD, Silva CS da, Araújo ACS, Vianna EO: **Development and validation of an asthma knowledge questionnaire for use in Brazil.** *J Bras Pneumol publicaçäo Of da Soc Bras Pneumol e Tisilogia* , **36**:8–13.

10. Bornovalova MA, Hicks BM, Patrick CJ, Iacono WG, McGue M: **Development and validation of the Minnesota borderline personality disorder scale.** *Assessment* 2011, **18**:234–52.

11. Boswell-Ruys CL, Harvey L a, Delbaere K, Lord SR: **A Falls Concern Scale for people with spinal cord injury (SCI-FCS).** *Spinal Cord* 2010, **48**:704–9.

12. Bowling A: **The Psychometric Properties of the Older People's Quality of Life Questionnaire, Compared with the CASP-19 and the WHOQOL-OLD.** *Curr Gerontol Geriatr Res* 2009, **2009**:12.

13. Brose LS, Bradley C: **Psychometric development of the individualized Retinopathy-Dependent Quality of Life Questionnaire (RetDQoL).** *Value Health* 2010, **13**:119–27.

14. Broström A, Arestedt KF, Nilsen P, Strömberg A, Ulander M, Svanborg E: **The side-effects to CPAP treatment inventory: the development and initial validation of a new tool for the measurement of side-effects to CPAP treatment.** *J Sleep Res* 2010, **19**:603–11.

15. Brown KF, Shanley R, Cowley NAL, van Wijgerden J, Toff P, Falconer M, Ramsay M, Hudson MJ, Green J, Vincent CA, Kroll JS, Fraser G, Sevdalis N: **Attitudinal and demographic predictors of measles, mumps and rubella (MMR) vaccine acceptance: development and validation of an evidence-based measurement instrument.** *Vaccine* 2011, **29**:1700–9.

16. Carvalho JP, Gawrysiak MJ, Hellmuth JC, McNulty JK, Magidson JF, Lejuez CW, Hopko DR: **The reward probability index: design and validation of a scale measuring access to environmental reward.** *Behav Ther* 2011, **42**:249–62.

17. Cheng WLS, Lai CKY: **Satisfaction Scale for Community Nursing: development and validation.** *J Adv Nurs* 2010, **66**:2331–40.

18. Cochrane GM, Marella M, Keeffe JE, Lamoureux EL: **The Impact of Vision Impairment for Children (IVI_C): validation of a vision-specific pediatric quality-of-life questionnaire using Rasch analysis.** *Invest Ophthalmol Vis Sci* 2011, **52**:1632–40.

19. Corona J, Matsumoto H, Roye DP, Vitale MG: **Measuring quality of life in children with early onset scoliosis: development and initial validation of the early onset scoliosis questionnaire.** *J Pediatr Orthop* 2011, **31**:180–5.

20. Dalton EJ, Rasmussen VN, Classen CC, Grumann M, Palesh OG, Zarcone J, Kraemer HC, Kirshner JJ, Colman LK, Morrow GR, Spiegel D: **Sexual Adjustment and Body Image Scale (SABIS): a new measure for breast cancer patients.** *Breast J* 2009, **15**:287–90.

21. Deal LS, DiBenedetti DB, Williams VS, Fehnel SE: **The development and validation of the daily electronic Endometriosis Pain and Bleeding Diary.** *Health Qual Life Outcomes* 2010, **8**:64.

22. Deal LS, Williams VSL, DiBenedetti DB, Fehnel SE: **Development and psychometric evaluation of the Endometriosis Treatment Satisfaction Questionnaire.** *Qual Life Res* 2010, **19**:899–905.

23. Doward LC, McKenna SP, Meads DM, Kahler K, Frech F: **The development of the Herpes Symptom Checklist and the Herpes Outbreak Impact Questionnaire.** *Value Health* 2009, **12**:139–45.

24. Ebrahimi N, Vohra-Miller S, Koren G: **Anorectal symptom management in pregnancy: development of a severity scale.** *J Popul Ther Clin Pharmacol* 2011, **18**:e99–e105.

25. Esplen MJ, Stuckless N, Berk T, Butler K, Gallinger S: **The FAP self-concept scale (adult form).** *Fam Cancer* 2009, **8**:39–50.

26. Fairchild H, Cooper M: **A multidimensional measure of core beliefs relevant to eating disorders: preliminary development and validation.** *Eat Behav* 2010, **11**:239–46.

27. Flokstra-de Blok BMJ, DunnGalvin A, Vlieg-Boerstra BJ, Oude Elberink JNG, Duiverman EJ, Hourihane JO, Dubois AEJ: **Development and validation of the self-administered Food Allergy Quality of Life Questionnaire for adolescents.** *J Allergy Clin Immunol* 2008, **122**:139–44, 144.e1–2.

28. Franzke N, Schäfer I, Jost K, Blome C, Rustenbach SJ, Reich K, Reusch M, Maurer M, Augustin M: **A new instrument for the assessment of patient-defined benefit in the treatment of allergic rhinitis.** *Allergy* 2011, **66**:665–70.

29. Garratt AM, Bjertnaes OA, Holmboe O, Hanssen-Bauer K: **Parent experiences questionnaire for outpatient child and adolescent mental health services (PEQ-CAMHS Outpatients): reliability and validity following a national survey.** *Child Adolesc Psychiatry Ment Health* 2011, **5**:18.

30. Ghisi GL de M, Durieux A, Manfroi WC, Herdy AH, Carvalho T de, Andrade A, Benetti M: **[Construction and validation of the CADE-Q for patient education in cardiac rehabilitation programs].** *Arq Bras Cardiol* 2010, **94**:813–22.

31. Goffaux P, Boudrias M, Mathieu D, Charpentier C, Veilleux N, Fortin D: **Development of a concise QOL questionnaire for brain tumor patients.** *Can J Neurol Sci* 2009, **36**:340–8.

32. Goldzweig G, Hasson-Ohayon I, Meirovitz A, Braun M, Hubert A, Baider L: **Agents of support: psychometric properties of the Cancer Perceived Agents of Social Support (CPASS) questionnaire.** *Psychooncology* 2010, **19**:1179–86.

33. Graesslin O, Barjot P, Hoffet M, Cohen D, Vaillant P, Clerson P: **The EVAPIL scale, a new tool to assess tolerance of oral contraceptives.** *Contraception* 2009, **80**:540–54.

34. Guex J-J, Rahhali N, Taïeb C: **The patient's burden of chronic venous disorders: construction of a questionnaire.** *Phlebology* 2010, **25**:280–5.

35. Hatt SR, Leske DA, Bradley EA, Cole SR, Holmes JM: **Development of a quality-of-life questionnaire for adults with strabismus.** *Ophthalmology* 2009, **116**:139–144.e5.

36. He Q, Feng Y, Wang Y, Wang Z: **Development and evaluation of the heroin abstainers' cue-sensitization questionnaire.** *J Huazhong Univ Sci Technolog Med Sci* 2009, **29**:134–8.

37. Helbostad JL, Oldervoll LM, Fayers PM, Jordhøy MS, Fearon KCH, Strasser F, Kaasa S: **Development of a computer-administered mobility questionnaire.** *Support Care Cancer* 2011, **19**:745–55.

38. Hildebrandt T, Walker DC, Alfano L, Delinsky S, Bannon K: **Development and validation of a male specific body checking questionnaire.** *Int J Eat Disord* 2010, **43**:77–87.

39. Hing N, Haw J: **The Development of a Multi-dimensional Gambling Accessibility Scale.** *J Gambl Stud* 2009:569–581.

40. Horvat N, Kos M: **Development and initial validation of a patient satisfaction with pharmacy performance questionnaire (PSPP-Q).** *Eval Health Prof* 2010, **33**:197–215.

41. Huguet A, McGrath PJ, Pardos J: **Development and preliminary testing of a scale to assess pain-related fear in children and adolescents.** *J Pain* 2011, **12**:840–8.

42. Jacob KL, Christopher MS, Neuhaus EC: **Development and validation of the cognitive-behavioral therapy skills questionnaire.** *Behav Modif* 2011, **35**:595–618.

43. Jessup MA, Dibble SL: **Validity and reliability of the COJAC screening tool for co-occurring disorders.** *Am J Addict* 2011, **20**:264–70.

44. Jones GL, Morrell CJ, Cooke JM, Speier D, Anumba D, Stewart-Brown S: **The development of two postnatal health instruments: one for mothers (M-PHI) and one for fathers (F-PHI) to measure health during the first year of parenting.** *Qual Life Res* 2011, **20**:1011–22.

45. Jones MP, Keefer L, Bratten J, Taft TH, Crowell MD, Levy R, Palsson O: **Development and initial validation of a measure of perceived stigma in irritable bowel syndrome**. *Psychol Health Med* 2009, **14**:367–374.

46. Justícia JL, Baró E, Cardona V, Guardia P, Ojeda P, Olaguíbel JM, Vega JM, Vidal C: **Development of a questionnaire to assess patient satisfaction with allergen-specific immunotherapy in adults: item generation, item reduction, and preliminary validation.** *Patient Prefer Adherence* 2011, **5**:239–50.

47. Kalf JG, Borm GF, de Swart BJ, Bloem BR, Zwarts MJ, Munneke M: **Reproducibility and validity of patient-rated assessment of speech, swallowing, and saliva control in Parkinson's disease.** *Arch Phys Med Rehabil* 2011, **92**:1152–8.

48. Keefer L, Kiebles JL, Taft TH: **The role of self-efficacy in inflammatory bowel disease management: preliminary validation of a disease-specific measure.** *Inflamm Bowel Dis* 2011, **17**:614–20.

49. Khadka J, Ryan B, Margrain TH, Court H, Woodhouse JM: **Development of the 25-item Cardiff Visual Ability Questionnaire for Children (CVAQC).** *Br J Ophthalmol* 2010, **94**:730–5.

50. Kizhner V, Xu D, Krespi YP: **A new tool measuring oral malodor quality of life.** *Eur Arch Otorhinolaryngol* 2011, **268**:1227–32.

51. Kripalani S, Risser J, Gatti ME, Jacobson TA: **Development and evaluation of the Adherence to Refills and Medications Scale (ARMS) among low-literacy patients with chronic disease.** *Value Health* 2009, **12**:118–23.

52. Krysa J, Lyons M, Williams AB: **A simple quality of life questionnaire for patients with faecal incontinence.** *Int J Colorectal Dis* 2009, **24**:1213–7.

53. Lai PSM, Chua SS, Chan SP, Low WY, Wong ICK: **Development and validation of the osteoporosis patient satisfaction questionnaire (OPSQ).** *Maturitas* 2010, **65**:55–63.

54. Larios SE, Ayala GX, Arredondo EM, Baquero B, Elder JP: **Development and validation of a scale to measure Latino parenting strategies related to children's obesigenic behaviors. The parenting strategies for eating and activity scale (PEAS).** *Appetite* 2009, **52**:166–72.

55. Lee CM, Neighbors C, Hendershot CS, Grossbard JR: **Development and preliminary validation of a comprehensive marijuana motives questionnaire.** *J Stud Alcohol Drugs* 2009, **70**:279–87.

56. Linden M, Baumann K, Lieberei B, Rotter M: **The Post-Traumatic Embitterment Disorder Self-Rating Scale (PTED Scale).** *Clin Psychol Psychother* 2009, **16**:139–47.

57. Linsell L, Forbes LJL, Burgess C, Kapari M, Thurnham A, Ramirez AJ: **Validation of a measurement tool to assess awareness of breast cancer.** *Eur J Cancer* 2010, **46**:1374–81.

58. Lo J-L, Yao G, Wang T-M: **Development of the Chinese language paediatric daily occupation scale in Taiwan.** *Occup Ther Int* 2010, **17**:20–8.

59. Løvås K, Curran S, Oksnes M, Husebye ES, Huppert FA, Chatterjee VKK: **Development of a disease-specific quality of life questionnaire in Addison's disease.** *J Clin Endocrinol Metab* 2010, **95**:545–51.

60. Mancuso CA, Sayles W, Allegrante JP: **Development and testing of the Asthma Self-Management Questionnaire.** *Ann Allergy Asthma Immunol* 2009, **102**:294–302.

61. Maples P, Franks A, Ray S, Stevens AB, Wallace LS: **Development and validation of a low-literacy Chronic Obstructive Pulmonary Disease knowledge Questionnaire (COPD-Q).** *Patient Educ Couns* 2010, **81**:19–22.

62. McCracken LM, Gauntlett-Gilbert J: **Role of psychological flexibility in parents of adolescents with chronic pain: development of a measure and preliminary correlation analyses.** *Pain* 2011, **152**:780–5.

63. McKenna SP, Meads DM, Doward LC, Twiss J, Pokrzywinski R, Revicki D, Hunter CJ, Glendenning GA: **Development and validation of the living with chronic obstructive pulmonary disease questionnaire.** *Qual Life Res* 2011, **20**:1043–52.

64. Merz RI, Deakin J, Hawthorne MR: **Oromandibular dystonia questionnaire (OMDQ-25): a valid and reliable instrument for measuring health-related quality of life.** *Clin Otolaryngol* 2010, **35**:390–6.

65. Mezzich JE, Cohen NL, Ruiperez MA, Banzato CEM, Zapata-Vega MI: **The Multicultural Quality of Life Index: presentation and validation.** *J Eval Clin Pract* 2011, **17**:357–64.

66. Mills RJ, Young CA, Pallant JF, Tennant A: **Development of a patient reported outcome scale for fatigue in multiple sclerosis: The Neurological Fatigue Index (NFI-MS).** *Health Qual Life Outcomes* 2010, **8**:22.

67. Mitchell AE, Fraser JA: **Parents' self-efficacy, outcome expectations, and self-reported task performance when managing atopic dermatitis in children: instrument reliability and validity.** *Int J Nurs Stud* 2011, **48**:215–26.

68. Montero J, Bravo M, López-Valverde A: **Development of a specific indicator of the well-being of wearers of removable dentures.** *Community Dent Oral Epidemiol* 2011, **39**:515–24.

69. Moscovitch DA, Huyder V: **The negative self-portrayal scale: development, validation, and application to social anxiety.** *Behav Ther* 2011, **42**:183–96.

70. Mullens AB, Young RM, Dunne M, Norton G: **The Cannabis Expectancy Questionnaire for Men who have Sex with Men (CEQ-MSM): A measure of substance-related beliefs.** *Addict Behav* 2010, **35**:616–9.

71. Murphy BA, Dietrich MS, Wells N, Dwyer K, Ridner SH, Silver HJ, Gilbert J, Chung CH, Cmelak A, Burkey B, Yarbrough WG, Sinard R, Netterville J: **Reliability and validity of the Vanderbilt Head and Neck Symptom Survey: a tool to assess symptom burden in patients treated with chemoradiation.** *Head Neck* 2010, **32**:26–37.

72. Nagpal J, Kumar A, Kakar S, Bhartia A: **The development of 'Quality of Life Instrument for Indian Diabetes patients (QOLID): a validation and reliability study in middle and higher income groups.** *J Assoc Physicians India* 2010, **58**:295–304.

73. Nagy Z, Bálint Z, Farkas H, Radics J, Kumánovics G, Minier T, Varjú C, Czirják L: **Establishment and partial validation of a patient skin self-assessment questionnaire in systemic sclerosis.** *Rheumatology (Oxford)* 2009, **48**:309–14.

74. Niedermann K, Forster A, Ciurea A, Hammond A, Uebelhart D, de Bie R: **Development and psychometric properties of a joint protection self-efficacy scale.** *Scand J Occup Ther* 2011, **18**:143–52.

75. Osman A, Gutierrez PM, Barrios F, Wong JL, Freedenthal S, Lozano G: **Development and initial psychometric properties of the University of Texas at San Antonio Future Disposition Inventory.** *J Clin Psychol* 2010, **66**:410–29.

76. Pakhale S, Wood-Dauphinee S, Spahija J, Collet J-P, Maltais F, Bernard S, Baltzan M, Rouleau M, Bourbeau J: **Combining both generic and disease-specific properties: development of the McGill COPD quality of life questionnaire.** *COPD* 2011, **8**:255–63.

77. Panagakou SG, Theodoridou MN, Papaevangelou V, Papastergiou P, Syrogiannopoulos GA, Goutziana GP, Hadjichristodoulou CS: **Development and assessment of a questionnaire for a descriptive cross-sectional study concerning parents' knowledge, attitudes and practises in antibiotic use in Greece.** *BMC Infect Dis* 2009, **9**:52.

78. Paranjape A, Rodríguez M, Gaughan J, Kaslow NJ: **Psychometric properties of a new scale to assess family violence in older African American women: The Family Violence Against Older Women (FVOW) Scale.** *Violence Against Women* 2009, **15**:1213–26.

79. Patrick DL, Edwards TC, Skalicky AM, Schick B, Topolski TD, Kushalnagar P, Leng M, O'Neill-Kemp AM, Sie KS: **Validation of a quality-of-life measure for deaf or hard of hearing youth.** *Otolaryngol Head Neck Surg* 2011, **145**:137–45.

80. Pokrzywinski RF, Meads DM, McKenna SP, Glendenning GA, Revicki DA: **Development and psychometric assessment of the COPD and Asthma Sleep Impact Scale (CASIS).** *Health Qual Life Outcomes* 2009, **7**:98.

81. Pusic AL, Klassen AF, Scott AM, Klok JA, Cordeiro PG, Cano SJ: **Development of a new patient-reported outcome measure for breast surgery: the BREAST-Q.** *Plast Reconstr Surg* 2009, **124**:345–53.

82. Riordain RN, McCreary C: **Validity and reliability of a newly developed quality of life questionnaire for patients with chronic oral mucosal diseases.** *J Oral Pathol Med* 2011, **40**:604–9.

83. Roberts ME, Barthel FM-S, Lopez C, Tchanturia K, Treasure JL: **Development and validation of the Detail and Flexibility Questionnaire (DFlex) in eating disorders.** *Eat Behav* 2011, **12**:168–74.

84. Roohafza H, Ramezani M, Sadeghi M, Shahnam M, Zolfagari B, Sarafzadegan N: **Development and validation of the stressful life event questionnaire.** *Int J Public Health* 2011, **56**:441–8.

85. Rosenberg L, Jarus T, Bart O: **Development and initial validation of the Children Participation Questionnaire (CPQ).** *Disabil Rehabil* 2010, **32**:1633–44.

86. Ruiz MA, Heras F, Alomar A, Conde-Salazar L, de la Cuadra J, Serra E, Regalado F, Halbach R: **Development and validation of a questionnaire on "Satisfaction with dermatological treatment of hand eczema" (DermaSat).** *Health Qual Life Outcomes* 2010, **8**:127.

87. Ruscheweyh R, Marziniak M, Stumpenhorst F, Reinholz J, Knecht S: **Pain sensitivity can be assessed by self-rating: Development and validation of the Pain Sensitivity Questionnaire.** *Pain* 2009, **146**:65–74.

88. Scoffier S, Paquet Y, Corrion K, d'Arripe-Longueville F: **Development and validation of the French Self-Regulatory Eating Attitude in Sports Scale.** *Scand J Med Sci Sports* 2010, **20**:696–705.

89. Sekiguchi M, Wakita T, Otani K, Onishi Y, Fukuhara S, Kikuchi S, Konno S: **Development and validation of a symptom scale for lumbar spinal stenosis.** *Spine (Phila Pa 1976)* 2012, **37**:232–9.

90. Shaikh N, Hoberman A, Paradise JL, Wald ER, Switze GE, Kurs-Lasky M, Colborn DK, Kearney DH, Zoffel LM: **Development and preliminary evaluation of a parent-reported outcome instrument for clinical trials in acute otitis media.** *Pediatr Infect Dis J* 2009, **28**:5–8.

91. Simard S, Savard J: **Fear of Cancer Recurrence Inventory: development and initial validation of a multidimensional measure of fear of cancer recurrence.** *Support Care Cancer* 2009, **17**:241–51.

92. Skogstad L, Hem E, Sandvik L, Ekeberg Ø: **The casualty chain inventory: a new scale for measuring peritraumatic responses: a cross-sectional study.** *BMC Emerg Med* 2011, **11**:6.

93. Smidt AC, Lai J-S, Cella D, Patel S, Mancini AJ, Chamlin SL: **Development and validation of Skindex-Teen, a quality-of-life instrument for adolescents with skin disease.** *Arch Dermatol* 2010, **146**:865–9.

94. Spangler DL: **The Change in Eating Disorder Symptoms scale: scale development and psychometric properties.** *Eat Behav* 2010, **11**:131–7.

95. Spreng RN, McKinnon MC, Mar RA, Levine B: **The Toronto Empathy Questionnaire: scale development and initial validation of a factor-analytic solution to multiple empathy measures.** *J Pers Assess* 2009, **91**:62–71.

96. St Louis BL, Firestone AR, Johnston W, Shanker S, Vig KWL: **Prospective patients rate practice factors: development of a questionnaire.** *Am J Orthod Dentofacial Orthop* 2011, **139**:235–41.

97. Stuge B, Garratt A, Krogstad Jenssen H, Grotle M: **The pelvic girdle questionnaire: a condition-specific instrument for assessing activity limitations and symptoms in people with pelvic girdle pain.** *Phys Ther* 2011, **91**:1096–108.

98. Sukhawarn R, Wiratchai N, Tatsanavivat P, Pitiyanuwat S, Kanato M, Srivannaboon S, Guyatt GH: **Development of a refractive error quality of life scale for Thai adults (the REQ-Thai).** *J Med Assoc Thai* 2011, **94**:978–84.

99. Tasca GA, Illing V, Balfour L, Krysanski V, Demidenko N, Nowakowski J, Bissada H: **Psychometric properties of self-monitoring of eating disorder urges among treatment seeking women: ecological momentary assessment using a daily diary method.** *Eat Behav* 2009, **10**:59–61.

100. Tickner S, Leman PJ, Woodcock A: **Design and validation of the Satisfaction With Immunisation Service Questionnaire (SWISQ).** *Vaccine* 2010, **28**:5883–90.

101. Tiffin PA, Kaplan C, Place M: **Brief report: Development of the family perceptions scale; a novel instrument for evaluating subjective functioning in the families of adolescents.** *J Adolesc* 2011, **34**:593–7.

102. Tinelli M, Blenkinsopp A, Bond C: **Development, validation and application of a patient satisfaction scale for a community pharmacy medicines-management service.** *Int J Pharm Pract* 2011, **19**:144–55.

103. Trego LL: **Development of the Military Women's Attitudes Toward Menstrual Suppression Scale: from construct definition to pilot testing.** *J Nurs Meas* 2009, **17**:45–72.

104. Tsai T-I, Lee S-YD, Tsai Y-W, Kuo KN: **Methodology and validation of health literacy scale development in Taiwan.** *J Health Commun* 2011, **16**:50–61.

105. Vinik E, Carlton CA, Silva MP, Vinik AI: **Development of the Norfolk quality of life tool for assessing patients with neuroendocrine tumors.** *Pancreas* 2009, **38**:e87–95.

106. Von Mackensen S, Czepa D, Herbsleb M, Hilberg T: **Development and validation of a new questionnaire for the assessment of subjective physical performance in adult patients with haemophilia--the HEP-Test-Q.** *Haemophilia* 2010, **16**:170–8.

107. Wan C, Fang J, Yang Z, Zhang C, Luo J, Meng Q, Jiang D: **Development and validation of a quality of life instrument for patients with liver cancer QOL-LC.** *Am J Clin Oncol* 2010, **33**:448–55.

108. Wang JY, Hart SL, Lee J, Berian JR, McCrea GL, Varma MG: **A valid and reliable measure of constipation-related quality of life.** *Dis Colon Rectum* 2009, **52**:1434–42.

109. Wicks P, Massagli M, Kulkarni A, Dastani H: **Use of an online community to develop patient-reported outcome instruments: the Multiple Sclerosis Treatment Adherence Questionnaire (MS-TAQ).** *J Med Internet Res* 2011, **13**:e12.

110. Williams AM, Kristjanson LJ: **Emotional care experienced by hospitalised patients: development and testing of a measurement instrument.** *J Clin Nurs* 2009, **18**:1069–77.

111. Wu L-M, Chin C-C, Chen C-H, Lai F-C, Tseng Y-Y: **Development and validation of the paediatric cancer coping scale.** *J Adv Nurs* 2011, **67**:1142–51.

112. Yap P, Luo N, Ng WY, Chionh HL, Lim J, Goh J: **Gain in Alzheimer care INstrument--a new scale to measure caregiving gains in dementia.** *Am J Geriatr Psychiatry* 2010, **18**:68–76.

113. Zeller MH, Modi AC: **Development and initial validation of an obesity-specific quality-of-life measure for children: sizing me up.** *Obesity (Silver Spring)* 2009, **17**:1171–7.

114. Zhang X, Wang A: **Development of a psychosocial adaptation questionnaire for Chinese patients with visual impairments.** *J Clin Nurs* 2011, **20**:2822–9.
