# Supplementary material for: Sample size used to validate a scale: a review of publications on newly-developed patient reported outcomes measures
Source: Health Qual Life Outcomes. 2014 Dec 9;12:2. doi: 10.1186/s12955-014-0176-2 (PMC4275948; doi:10.1186/s12955-014-0176-2)
Supplement: Additional file 2: — Practices of the psychometric properties’ evaluation. Tables. [file 12955_2014_176_MOESM2_ESM.pdf]

How many individuals are used to validate a scale? A review of publications on newly-developed patient reported outcomes measures

Emmanuelle Anthoine, Leïla Moret, Antoine Regnault, Véronique Sébille, Jean-Benoît Hardouin

<u>Practices of the psychometric properties' evaluation</u>

Table 1: Information collected in articles

1 – General information	Title, country, journal, kind of journal, impact factor and year of publication
2 – Study and scale	Concept of interest, primary psychometric validation study of the scale: main or ancillary objective of the study.<br>Language of the scale development, number of items, number of factors, number of items per factor, kind of measurement scale, computation of a global score, computation of a score per factor, generic or specific scale.
3 – Sample size determination	How was sample size determined? How was it computed?<br>References used and authors' comments on the sample size in the discussion.
4 – Item distribution	Computation of missing value rates, how missing values are handled? Were Items eliminated when missing values rate was high? How high? Distribution of items or scores, ceiling and floor effects of items or scores.
5 – Psychometric properties	Content validity, face validity, construct validity (known group validity, convergent validity, divergent validity), reliability (internal consistency and repeatability), criterion validity (concurrent validity and predictive validity), responsiveness and Item Response Theory (IRT) model.

Table 2: Distribution of scores and items responses

n = 114		
Evaluation of ceiling and floor effect		35.1% (40)
Displaying of items or scores distribution		61.4% (70)
Description of items or scores missing value rate		21.9% (25)
Elimination of items based on missing value rate (n=25)		32% (8)
	Mean missing value rate* (SD); range	18% (7.9%) ; [10% ; 33%]

Data are percentages (n) and otherwise indicated. *Mean of the threshold used to eliminate the items.

Table 3: Scoring

n = 114		
Score per dimension	Yes	65.8% (75)
	No	12.3% (14)
	Not applicable (unidimensional scale)	21.9% (25)
Scoring	Sum	33.3% (38)
	Mean	8.8% (10)
	Linear transformation (between 0 and 1 or 10 or 100)	14.0% (16)
	Not mentioned	43.8% (50)

Table 4: Assessment of validity

n = 114		
**Content validity***		94.7% (108)
Literature review (n=97)		75.2% (73)
Patient focus group (n = 97)		22.7% (22)
Sample size	Mean (SD) ; Median ; range	36.7 (27.4) ; 30 ; [10 ; 98]
Patient Interviews (n = 97)		37.1% (36)
Sample size	Mean (SD) ; Median ; range	37.6 (76.5) ; 22 ; [7 ; 422]
Experts (n=97)		78.3% (76)
**Face validity***		65.8% (75)
Pilot testing on a small sample (n = 70)		100% (70)
Sample size	Mean (SD) ; Median ; range	54.2 (97.9) ; 20 ; [3 ; 603]
Understanding of items (n=70)		97.1% (68)
Item refinement (n = 70)		85.7% (60)
**Criterion validity***		70.2% (80)
Concurrent validity (n=80)		98.7% (79)
Predictive validity (n=80)		3.7% (3)
**Construct validity***		90.3% (103)
Convergent validity (n = 103)		84.5% (87)
Divergent validity (n = 103)	Yes	17.5% (18)
	No	63.1% (65)
	Not applicable (unidimensional scale)	19.4% (20)
Known-group validity (n = 103)		57.3% (59)
Exploratory factor analysis (n = 103)		79.6% (82)
Confirmatory factor analysis (n = 103)		15.5% (16)
**IRT model**		14.0% (16)
	Rasch modelling	12.2% (14)
	Other	1.7% (2)

Data are percentages (n) and otherwise indicated. *At least one aspect of a measurement property evaluating content, face, criterion or construct validity.

Table 5: Exploratory and confirmatory factor analysis

n = 103		
**Exploratory factor analysis**		79.6% (82)
Threshold factor loadings (n = 82)	> 0.4 without considering cross loadings	25.6% (21)
	> another threshold (between 0.3 and 0.6) without considering cross loadings	23.2% (19)
	Considering cross loadings	19.5% (16)
	Not mentioned	31.7% (26)
Rotation (n = 82)	Varimax	53.7% (44)
	Promax	12.2% (10)
	Other	15.9% (13)
	None or not mentioned	18.2% (15)
	Method justified (n=67)	22.4% (15)
% global variance explained	Mean (SD) ; Median ; range	61.1 (12.0) ; 61 ; [32.5 ; 88]
**Confirmatory factor analysis**		15.5% (16)
Definition of the structure (n=16)	EFA	87.5% (14)
	Qualitative analysis	12.5% (2)
Fit indices* (n=16)	RMSEA	93.7% (15)
	CFI	81.2% (13)
	Khi2	75.0% (12)
	TLI	43.7% (7)
	SRMR	31.2% (5)
	GFI	31.2% (5)
	AGFI	31.2% (5)
	Other	100.0%(16)

Data are percentages (n) and otherwise indicated. *Several coefficients could be used.

Table 6: Assessment of reliability and responsiveness

n = 114		
**Repeatability**		60.5% (69)
Sample size	Mean (SD) ; Median ; range	85 (86) ; 53 ; [8 ; 491]
Determination of the required sample size (n=69)		0.0% (0)
Coefficient* (n = 69)	ICC	52.2% (36)
	Correlation coefficient	49.3% (34)
	Paired t test	10.1% (7)
	Other	11.6% (8)
**Internal consistency**		89.5% (102)
Coefficient* (n = 102)	Cronbach alpha	95.1% (97)
	Inter item correlation	10.8% (11)
	Split half	6.8% (7)
	Other	5.9% (6)
**Responsiveness**		10.5% (12)
Coefficient* (n = 12)	Paired t test	75.0% (9)
	Effect Size	25.0% (3)
	Standarized Response Mean	16.7% (2)
	Responsiveness statistic	0.0% (0)
	Other	16.7% (2)

Data are percentages (n) and otherwise indicated. *Several coefficients could be used.
